# Sickness among Animals

**Published:** 1919-11-01

**Authors:** 


### SICKNESS AMONG ANIMALS.

Realisation of the important and direct part played by animals in the transference and dissemination of disease, and the close association in which so many of them live with human beings, should make our health authorities take even more care to bring home to the laity the enormously important bearing which the proper health and supervision of domesticated beasts have upon our own well-being. Periodically a great crusade is made to defeat the baneful influence of some common insect pest, or to bring more careful observation to bear upon our food articles of animal origin, yet, if one can judge from conversations to be heard every day, there is very little general distribution of elementary knowledge on many of the commonest pathological conditions in animals, and the bad effect they may directly produce upon human health. It is considered of many conditions which may occur in a sick animal that they cannot be directly transferred to man, yet it is certain that they can infect him, although they are never seen to produce the same lesions as in the beast. The infecting organisms, be they simple pyogenic cocci or more specialised types, can do untold harm in a community. In future issues of this journal we hope to draw more detailed attention to some outstanding instances of this menace, but must at present confine our remarks to the recently published report of the Chief Veterinary Officer, as giving valuable indication of the situation as it stands at the opening of the country's health campaign.

In many respects the report is highly satisfactory. The unusual rural conditions, the shortage of farm attendants, and the service importation and later demobilisation of so many animals, might well have been expected to produce a great increase and wider distribution of diseases to which they were liable, but on the whole this has not occurred.

To take some figures in illustration, in all during the year 1918 10,203 cases of swine fever were reported, but of these only 1,407—namely, 13.8 per cent.—were confirmed, and it is at least possible that, although the wish to act in accordance with the law is present, there is considerable need for the owners of these animals to recognise more accurately the signs of their many ailments. Glanders has frequently occurred, as usual mostly in the London area, but it is noteworthy that no outbreak has appeared among colliery horses. Although anthrax has of late been frequently discussed in connection with Asiatic hides, wool, etc., there is a great decrease in cases for the year in question. Scotland shows the greatest improvement, but it is suggested that this apparent immunity is largely due to the greatly restricted importations of feeding-stuffs, artificial manures, etc., prevailing at the time, and that we must expect a tendency to return to the pre-war prevalence. The risk in these feeding-stuffs is brought out by the fact that ninety-one out of 182 outbreaks during the year on previously clean premises were traced to this origin. The value of notification and double dipping of sheep is clearly proved, and similarly the local success and necessity for extension of a host of simple hygienic measures applied to animals in general.

To the lay reader the report may too greatly abound with technicalities, but, if so, we would at least refer him to the section dealing with rabies. The tracing of the early outbreaks, their successful counteraction, and the clear exposition of treatment organisation, requirements, and results, serve as an excellent model upon which to build our views and relative attitude towards infectious animal disease. True, we are in this instance dealing with a micro-organism deadly in the extreme, but let us realise that the multitude of common suppurations, parasitic diseases, or apparently simple ailments in the animals around us are of importance fully as vital to the health and welfare of the people, when their vastly greater prevalence is considered. Bye-laws, notifications and the rest have been invaluable means to an end, but we believe that yet further improvement may be expected to follow a more general education of the public in these matters.

